# Our Sick Soldiers

**Published:** 1863-01

**Authors:** 


### OUR SICK SOLDIERS.

Directory of the Hospitals.—The Sanitary Commission have established an office of information in regard to patients in the hospitals of the District of Columbia, and of Frederick City, Maryland. By a reference to books, which are corrected daily, an answer can, under ordinary circumstances, be given by return mail to the following questions:

1st. Is —— —— [giving name and regiment] at present in the hospitals of the District or of Frederick City?

2d. If so, what is his proper address?

3d. What is the name of the Surgeon or Chaplain of the hospital?

4th. If not in hospital at present, has he recently been in hospital?

5th. If so, did he die in hospital, and at what date?

6th. If recently discharged from hospital, was he discharged from service?

7th. If not, what were his orders on leaving?

The Commission is prepared also to furnish more specific information as to the condition of any patient in the District hospitals, within twenty-four hours after a request to do so, from an officer of any of its corresponding societies.

The office of the Directory will be open daily from 8 o'clock A.M. to 8 o'clock P.M., and accessible in urgent cases at any hour of the night.

The number of patients in these hospitals is about twenty-five thousand. If found to be practicable, the duty here undertaken locally by the Commission will be extended to include all the general hospitals in the country. FRED. LAW OLMSTEAD, *General Secretary.*

Adams House, No. 244 F street, *Washington, D. C., Nov. 19th*, 1862.

To Farmers.—That excellent weekly, *The New-England Farmer*, of Boston, says: "We learn through the newspapers that this gentleman has been appointed by Mr. Commissioner Newton to the chief clerkship in the Agricultural Department at Washington. We know Mr. Grennell well—know him in the social relations of life, and as connected with agriculture, theoretically and practically—having been associated with him in the Massachusetts State Board of Agriculture, where there were excellent opportunities to learn his tastes, powers, and energy in the great subject, and we do not hesitate to say that we believe the appointment a most judicious one. Mr. G. has youth, health, an ardent temperament, sound learning from books and institutions, together with untiring energy, integrity, and much personal acquaintance and experience on the farm—all of which combined give him qualifications for the position with which he has been intrusted, which few can expect to possess. We congratulate the Commissioner in his wise selection, and have no doubt but Mr. Grennell will relieve him of a vast amount of labor which might embarrass him in the general management of the Department."

Feet Diseases.—Prof. Cleaveland, of Cincinnati, has done the public an important service in issuing a paper-covered volume, sent post-paid, for fifty cents, on the causes and cure of diseases of the feet, with practical suggestions as to their clothing; it is the most complete, reliable, and useful volume which has appeared in this country on these subjects; no ailment of the feet has been omitted. The same industrious author has issued an admirable "Physician's Memorandum" for 1863, containing a list of remedial agents, their nature, doses, etc.; abbreviations, notes for accidents and emergencies, *post-mortem* examinations, preservation, embalmings, etc., etc.

Soldiers and Sailors.—Among the books and tracts published by The American Tract Society, at No. 28 Cornhill, Boston, and also at the Bible House, New-York City, the most beautiful in subject, execution, and embellishment are:

| | | | | | | | |
|---|---|---|---|---|---|---|---|
| The Cross-Bearer,..... | $1 50 | pp. | $1 70 | Little Captain,.......... | $0 25 | pp. | $0 31 |
| Uncle Paul's Stories,.. | 50 | " | 65 | Picture-Book,.......... | 25 | " | 30 |

Almanac for 1862,..... 00 06 pp. 00 07.

The Society have published for our brave soldiers in the army ten Pocket Tracts of sixteen pages each, for ten cents, entitled, "Take Care of your Health," "Rest," "The Widow's Son," etc. Also forty-eight Soldiers' Envelope Tracts, four pages each, price ten cents, suitable to be inclosed in envelopes when writing to soldiers, without increasing the letter-postage. Address J. G. Broughton, Depositary, 13 Bible House, New-York City. Also, "Hints to Soldiers for the Preservation of Health," with three excellent prayers at the end for morning, evening, and before going into battle; also "How to be Saved, in Three Letters to a Friend," by Francis Wayland; also a delightful exemplification of "Faithfulness," as illustrated in the life and labors of Rev. Morrison Huggins, by Rev. Charles P. Bush; also a most important 12mo, pp. 463, on the "Canon of the Holy Scriptures," examined in the light of history, by Prof. L. Gaussen, of Geneva, Switzerland, translated from the French and abridged by that able scholar and eloquent divine, Edward N. Kirk, D.D. It is a work which ably discusses a variety of points of the deepest interest to theological students and clergymen, and which ought to be in the library of every Bible-scholar.

